# Updates in Cardiac Amyloidosis: A Review

**DOI:** 10.1161/JAHA.111.000364

**Published:** 2012-04-24

**Authors:** Sanjay M. Banypersad, James C. Moon, Carol Whelan, Philip N. Hawkins, Ashutosh D. Wechalekar

**Affiliations:** National Amyloidosis Centre, UCL Medical School, UK (S.M.B., C.W., P.N.H., A.D.W.); The Heart Hospital, UK (S.M.B., J.C.M.); University College London, UK (S.M.B., C.W.); Institute of Cardiovascular Sciences, University College of London, UK (J.C.M.); Centre for Amyloidosis and Acute Phase Proteins, Division of Medicine, University College of London, UK (P.N.H., A.D.W.)

**Keywords:** cardiac amyloidosis, infiltrative cardiomypathy, treatment

## Introduction

Systemic amyloidosis is a relatively rare multisystem disease caused by the deposition of misfolded protein in various tissues and organs. It may present to almost any specialty, and diagnosis is frequently delayed.^1^ Cardiac involvement is a leading cause of morbidity and mortality, especially in primary light chain (AL) amyloidosis and in both wild-type and hereditary transthyretin amyloidosis. The heart is also occasionally involved in acquired serum amyloid A type (AA) amyloidosis and other rare hereditary types. Clinical phenotype varies greatly between different types of amyloidosis, and even the cardiac presentation has a great spectrum. The incidence of amyloidosis is uncertain, but it is thought that the most frequently diagnosed AL amyloidosis has an annual incidence of 6 to 10 cases per million population in the United Kingdom and United States. Amyloidosis due to transthyretin deposition (ATTR) can be wild-type transthyretin amyloid deposits, which predominantly accumulate in the heart and are very common at autopsy in the elderly. Although the associated clinical syndrome known as senile systemic amyloidosis is diagnosed rarely in life,^2^ there is increasing evidence that this disorder is much underdiagnosed and that with increasing longevity and improved diagnostic methods it may be identified as a substantial public health problem.

This review focuses on recent progress in the field: novel diagnostic and surveillance approaches using imaging (echocardiography, cardiovascular magnetic resonance), biomarkers (brain natriuretic peptide [BNP], high-sensitivity troponin), new histological typing techniques, and current and future treatments, including approaches directly targeting the amyloid deposits.^3^

## Pathophysiology

Amyloidosis is caused by the extracellular deposition of autologous protein in an abnormal insoluble β-pleated sheet fibrillary conformation—that is, as amyloid fibrils. More than 30 proteins are known to be able to form amyloid fibrils in vivo, which cause disease by progressively damaging the structure and function of affected tissues.^4^ Amyloid deposits also contain minor nonfibrillary constituents, including serum amyloid P component (SAP), apolipoprotein E, connective tissue components (glycosaminoglycans, collagen), and basement membrane components (fibronectin, laminin).^3,5–8^ Amyloid deposits can be massive, and cardiac or other tissues may become substantially replaced. Amyloid fibrils bind Congo red stain, yielding the pathognomonic apple-green birefringence under cross-polarized light microscopy that remains the gold standard for identifying amyloid deposits.

## Clinical Features

Cardiac amyloidosis, irrespective of type, presents as a restrictive cardiomyopathy characterized by progressive diastolic and subsequently systolic biventricular dysfunction and arrhythmia.^1^ Key “red flags” to possible systemic amyloidosis include nephrotic syndrome, autonomic neuropathy (eg, postural hypotension, diarrhea), soft-tissue infiltrations (eg, macroglossia, carpal tunnel syndrome, respiratory disease), bleeding (eg, cutaneous, such as periorbital, gastrointestinal), malnutrition/cachexia and genetic predisposition (eg, family history, ethnicity). Initial presentations may be cardiac, with progressive exercise intolerance and heart failure. Other organ involvement, particularly in AL amyloidosis, may cloud the cardiac presentation (eg, nephrotic syndrome, autonomic neuropathy, pulmonary or bronchial involvement). Pulmonary edema is not common early in the disease process,^9^ but pleural and pericardial effusions and atrial arrhythmias are often seen.^10,11^ Syncope is common and a poor prognostic sign.^12^ It is typically exertional or postprandial as part of restrictive cardiomyopathy, sensitivity to intravascular fluid depletion from loop diuretics combined with autonomic neuropathy, or conduction tissue involvement (atrioventricular or sinoatrial nodes) or ventricular arrhythmia.^13–15^ The latter may rarely cause recurrent syncope. Disproportionate septal amyloid accumulation mimicking hypertrophic cardiomyopathy with dynamic left ventricular (LV) outflow tract obstruction^16–19^ is rare but well documented. Myocardial ischemia can result from amyloid deposits within the microvasculature.^20,21^ Atrial thrombus is common, particularly in AL amyloidosis, sometimes before atrial fibrillation occurs.^22^ Intracardiac thrombus can embolize, causing transient ischemic attacks or strokes, and may be an early or even presenting feature.^23^ Anticoagulation is therefore important in the appropriate clinical situation, but careful consideration must be given to patients with extensive systemic AL amyloidosis who may have an elevated bleeding risk due to factor X deficiency or in some cases with gastrointestinal involvement.^24^ The Table gives an outline of the clinical phenotypes of the common amyloid subtypes.

### AL Amyloidosis

AL amyloidosis is caused by deposition of fibrils composed of monoclonal immunoglobulin light chains and is associated with clonal plasma cell or other B-cell dyscrasias. The spectrum and pattern of organ involvement is very wide, but cardiac involvement occurs in half of cases and is sometimes the only presenting feature.^25^ Cardiac AL amyloidosis may be rapidly progressive. Low QRS voltages, particularly in the limb leads, are common. Thickening of the LV wall is typically mild to moderate and is rarely >18 mm even in advanced disease. Cardiac AL amyloid deposition is accompanied by marked elevation of the biomarkers BNP and cardiac troponin, even at an early stage. Involvement of the heart is the commonest cause of death in AL amyloidosis and is a major determinant of prognosis; without cardiac involvement, patients with AL amyloidosis have a median survival of around 4 years,^26^ but the prognosis among affected patients with markedly elevated BNP and cardiac troponin (Mayo stage III disease)^27^ is on the order of 8 months.

### Hereditary Amyloidoses

Mutations in several genes, such as transthyretin, fibrinogen, apolipoprotein A1, and apolipoprotein A2 can be responsible for hereditary amyloidosis, but by far the most common cause is variant ATTR amyloidosis (variant ATTR) caused by mutations in the transthyretin gene causing neuropathy and, often, cardiac involvement.

The TTR gene is synthesized in the liver, and several point mutations are described (see the Table 1), but the most common is the Val122Ile mutation.^28,29^ In a large autopsy study that included individuals with cardiac amyloidosis, the TTR Val122Ile allele was present in 3.9% of all African Americans and 23% of African Americans with cardiac amyloidosis. Penetrance of the mutation is not truly known and is associated with a late-onset cardiomyopathy that is indistinguishable from senile cardiac amyloidosis. Although the prevalence of disease caused by this mutation is unknown, it is almost certainly underdiagnosed, because the wall thickening is often incorrectly attributed to hypertensive heart disease. Neuropathy is not generally a feature of this ATTR due to Val122Ile.

**Table.** Summary of Pathology, Presentation, and Management of Different Amyloid Types

Amyloid Type	Precursor Protein	Typical Decade of Presentation	Cardiac Involvement	Other Organ Involvement	Treatment	Prognosis (Median Survival)
Primary (AL) amyloidosis	Monoclonal light chain	6th or 7th decade (but can be any)	40% to 50%	Renal, liver, soft tissue, neuropathy	Chemotherapy or peripheral blood stem cell transplantation	48 mo but 8 mo for advanced-stage disease
Transthyretin amyloidosis						
ATTR (V30M)	Variant transthyretin	3rd or 4th decade (but geographical variation)	Uncommon (but can occur in older patients)	Peripheral and autonomic neuropathy	Liver transplantation (younger cases) not proven in others	Good with liver transplantation for V30M progressive disease
ATTR (T60 A)	Variant transthyretin	6th decade	Up to 90% by diagnosis	Peripheral and autonomic neuropathy	Liver transplantation possible in selected patients	Variable with liver transplantation
Wild-type ATTR	Wild-type transthyretin	70 y (but remains a consideration after 50 y)	Almost all cases	Carpal tunnel syndrome	Supportive	7 to 8 y
ATTR Ile 122	Variant transthyretin	6th decade or older	Almost all cases	Carpal tunnel syndrome	Supportive	7 to 8 y
Apolipoprotein A1 (ApoA1)	Variant apolipoprotein	6th decade or older	Rare	Predominantly renal	Renal (±liver) transplantation	Usually slowly progressive (y)
Secondary (AA) amyloidosis	Serum amyloid A (SAA)	Any	Rare	Renal, liver	Treat underlying inflammatory condition	Good
Atrial natriuretic peptide (ANP)	ANP	70 y or older	All cases but significance uncertain	None reported	Not needed	-

More than 100 genetic variants of TTR are associated with amyloidosis. Most present as the clinical syndrome of progressive peripheral and autonomic neuropathy. Unlike wild-type ATTR or variant ATTR Val122Ile, the features of other variant ATTR include vitreous amyloid deposits or, rarely, deposits in other organs. Cardiac involvement in variant ATTR varies by mutations and can be the presenting or indeed the only clinical feature.^30^ For example, cardiac involvement is rare in variant ATTR associated with Val30Met (a common variant in Portugal or Sweden), but it is almost universal and develops early in individuals with variant ATTR due to Thr60Ala mutation (a mutation common in Ireland).

Mutations in apolipoprotein A1 gene can cause systemic amyloidosis, typically causing renal and hepatic involvement—although cardiac involvement is well recognized.^31^

### Senile Systemic Amyloidosis (Wild-Type ATTR)

Wild-type TTR amyloid deposits are found at autopsy in about 25% of individuals >80 years of age, but their clinical significance has not been clear.^32–34^ The prevalence of wild-type TTR deposits leading to the clinical syndrome of wild-type ATTR cardiac amyloidosis remains to be ascertained, but the syndrome is distinct and clearly far rarer. Wild-type ATTR is a predominantly cardiac disease, and the only other significant extracardiac feature is a history of carpal tunnel syndrome, often preceding heart failure by 3 to 5 years.^35^ Extracardiac involvement is most unusual.

Both wild-type ATTR and ATTR due to Val122Ile are diseases of the >60-year age group and are often misdiagnosed as hypertensive heart disease.^9^ Wild-type ATTR has a strong male predominance, and the natural history remains poorly understood, but studies suggest a median survival of about 7 years from presentation.^32,33^ The true incidence of wild-type ATTR is probably underestimated, and recent developments in cardiac magnetic resonance (CMR), which have greatly improved detection of cardiac amyloid during life, suggest that wild-type ATTR is more common than previously thought: It accounted for 0.5% of all patients seen at the UK amyloidosis center until 2001 but now accounts for 7% of 1100 cases with amyloidosis seen since the end of 2009 (unpublished data). There appears to be an association between wild-type ATTR and history of myocardial infarctions, G/G (Val/Val) exon 24 polymorphism in the alpha2-macroglobulin (alpha2M), and the H2 haplotype of the tau gene^36^; the association of tau with Alzheimer's disease raises interesting questions as both are amyloid-associated diseases of aging. Although the echocardiographic manifestations of cardiac ATTR may be indistinguishable from advanced AL amyloidosis, patients with the former typically have fewer symptoms and better survival.^37^

### Other Types of Cardiac Amyloidosis

Localized atrial amyloid deposits derived from atrial natriuretic peptide are associated with atrial fibrillation, notably postoperatively,^9,38^ and become ubiquitous with age, being present at autopsy in 80% of people >70 years of age.^39,40^ The significance and causality of atrial natriuretic peptide amyloid deposits remain unknown. Amyloid of as yet unknown fibril type is also common in explanted cardiac valves.^41,42^ Systemic AA amyloidosis complicating chronic inflammatory diseases, in which the amyloid fibrils are derived from the acute-phase reactant serum amyloid A protein, involves the heart in about 2% of cases with systemic AA amyloidosis. Incidence of AA amyloidosis is generally in decline, likely reflecting better treatment for rheumatological disorders with biological agents.

## Diagnosis and Evaluation of Cardiac Amyloidosis

### Electrocardiography

**Figure 1.**
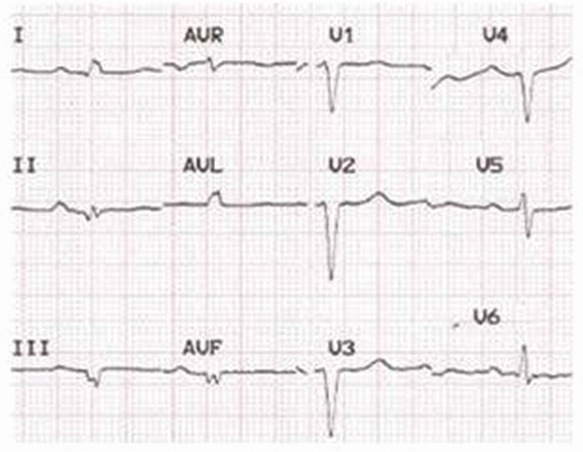
ECG of a patient with cardiac AL amyloidosis showing small QRS voltages (defined as ≤6 mm height), predominantly in the limb leads and pseudoinfarction pattern in the anterior leads.

Low QRS voltages (all limb leads <5 mm in height) with poor R-wave progression in the chest leads (pseudoinfarction pattern) occur in up to 50% of patients with cardiac AL amyloidosis.^43^ The combination of low ECG voltage with concentrically increased wall thickness is highly suspicious for cardiac amyloidosis (see Figure 1), but voltage criteria for LV hypertrophy can nevertheless sometimes occur.^44^ Other findings include first-degree atrioventricular block (21%), nonspecific intraventricular conduction delay (16%), second- or third-degree atrioventricular block (3%), atrial fibrillation/flutter (20%), and ventricular tachycardia (5%).^44^ Left and right bundle branch block can also occur.^18^ ECG patterns can provide clues to differentiate between AL and TTR amyloidosis: Left bundle branch block is seen in 40% of patients with wild-type ATTR but is rare in AL (4%), whereas typical low QRS voltages are seen in 40% wild-type ATTR versus 60% AL.^45^ There has been little recent study of ECG correlation with cardiac biomarkers, treatment toxicity, and mortality. Progressive ECG changes may be useful in assessing silent cardiac progression.^46^ Changes in ECG abnormalities after treatment in AL amyloidosis remain poorly studied but can occur—more often little improvement is seen. Holter ECG monitoring identifies asymptomatic arrhythmias in >75% of cardiac AL patients (mainly supraventricular tachyarrhythmias and some nonsustained ventricular tachycardia).^47^

### Echocardiography

All patients with suspected amyloidosis should undergo echocardiography. Findings are characteristic in advanced disease but are harder to elicit earlier on and have prognostic as well as diagnostic significance.^48–50^ Typical findings include concentric ventricular thickening with right ventricular involvement, poor biventricular long-axis function with normal/near-normal ejection fraction,^51,52^ and valvular thickening (particularly in wild-type or variant ATTR).^45^ Diastolic dysfunction is the earliest echocardiographic abnormality and may occur before cardiac symptoms develop.^53,54^ As with all investigations, echocardiography must be interpreted within the clinical context; a speckled or granular myocardial appearance, although characteristic of amyloid, is an inexact finding, which is dependent on machine gain settings. Biatrial dilatation in presence of biventricular, valvular, and interatrial septal thickening ^53^ is a useful clue to the diagnosis.

**Figure 2.**
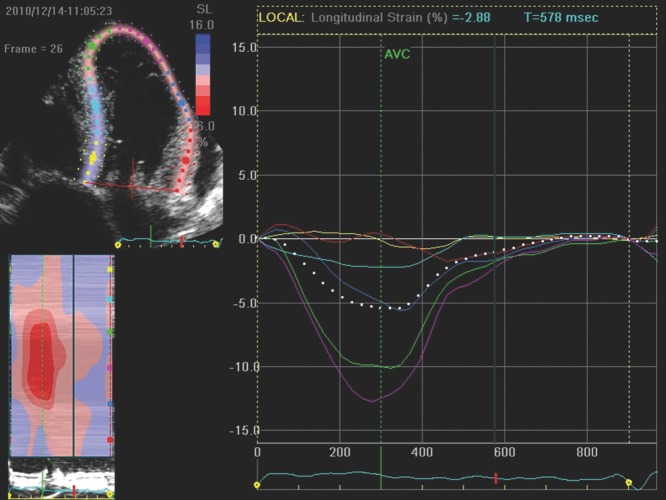
Transthoracic echocardiogram with speckle tracking. The red and yellow lines represent longitudinal motion in the basal segments, whereas the purple and green lines represent apical motion. This shows loss of longitudinal ventricular contraction at the base compared to apex.

Advanced echocardiographic techniques are beginning to reveal more about the underlying pathology and functional abnormalities, such as the twisting and untwisting cardiac motion that may be augmented through compensatory mechanisms before reversing to impairment later in the course of the disease.^55,56^ Strain and strain rate imaging, derived from speckle tracking (see Figure 2), may help differentiate cardiac amyloidosis from hypertrophic cardiomyopathy.^57,58^ Typically, there is much greater restriction of basal compared to apical movement. Mean LV basal strain is an independent predictor of both cardiac and overall deaths. Contrast echocardiography using transpulmonary bubble contrast can show microvascular dysfunction in AL amyloidosis.^59^ Although transesophageal echo may help detect atrial appendage thrombus in a third of cases of AL amyloid, translation of this into routine clinical practice for this frail and unwell patient population needs further study.^53,60^

### Cardiac Biomarkers

Measurements of BNP, its more stable N-terminal fragment (NT-proBNP), and cardiac troponins are extremely informative in AL amyloidosis, which is the only type in which they have been systematically studied to date. Their value in TTR amyloidosis is yet to be determined. BNP/NT-proBNP is cleared by the kidneys (BNP also partially cleared by the liver), confounding evaluation of patients with kidney involvement. Elevated NT-proBNP levels in systemic AL amyloidosis are a sensitive marker of cardiac involvement, with a cutoff >152 pmol/L being associated with higher mortality rate (72% vs 7.6% per year).^61^ Abnormal NT-proBNP is predictive of clinically significant cardiac involvement developing in the future.^62^ BNP/NT-proBNP in general reflects high filling pressures, but amyloid deposits may have a local effect—BNP granules are found in higher quantities in myocytes adjacent to amyloid deposits.^63^ Increased troponin concentrations are a marker of poor prognosis,^64^ but the mechanism remains unclear. High-sensitivity troponin is abnormal in >90% of cardiac AL patients,^65^ and the combination of BNP/NT-proBNP plus troponin measurements is used to stage and risk-stratify patients with AL amyloidosis at diagnosis.^27,66^ Very interestingly, the concentration of BNP/NT-proBNP in AL amyloidosis may fall dramatically within weeks after chemotherapy that substantially reduces the production of amyloidogenic light chains.^67^ The basis for this very rapid phenomenon, which is not mirrored by changes on echocardiography or CMR, remains uncertain, but a substantial fall is associated with improved outcomes. An early transient increase in BNP/NT-proBNP may occur after treatment with the immunomodulatory drugs thalidomide and lenalidomide, which are frequently used in the management of AL amyloidosis (see later), but the significance and cause are unclear.^68,69^

### Cardiac Magnetic Resonance

**Figure 3.**
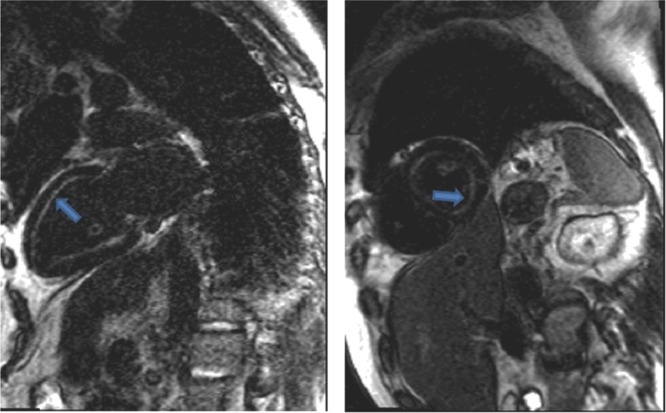
CMR with the classic amyloid global, subendocardial late gadolinium enhancement pattern in the left ventricle with blood and mid-/epimyocardium nulling together.

CMR provides functional and morphological information on cardiac amyloid in a similar way to echocardiography, though the latter is superior for evaluating and quantifying diastolic abnormalities. An advantage of CMR is in myocardial tissue characterization. Amyloidotic myocardium reveals subtle precontrast abnormalities (T1, T2),^70,71^ but extravascular contrast agents based on chelated gadolinium provide the key information. The appearance (see Figure 3) of global, subendocardial late gadolinium enhancement is highly characteristic of cardiac amyloid^72,73^ and correlates with prognosis.^74,75^ CMR is especially useful in patients with other causes of LV thickening/hypertrophy because it can differentiate amyloidosis from hypertension, which may not be possible by routine echocardiography.

**Figure 4.**
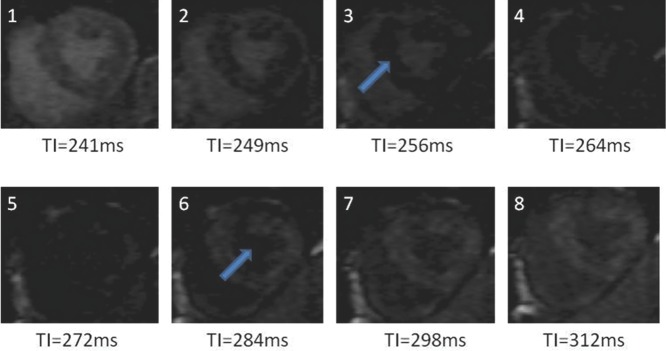
Sequential static images from a CMR TI scout sequence. As the inversion time (TI) increases, myocardium nulls first (arrow in image 3), followed by blood afterwards (arrow in image 6), implying that there is more gadolinium contrast in the myocardium than blood—a degree of interstitial expansion such that the “myocrit” is smaller than the hematocrit.

Difficulties are often encountered, however. For example, arrhythmias, particularly atrial fibrillation and ectopic beats, degrade image quality during CMR, and increasing experience of the technique in clinical practice has shown that the pattern of late gadolinium enhancement can be atypical and patchy, especially in early disease.^76^ Late gadolinium enhancement imaging in amyloidosis is inherently challenging because amyloid infiltration within the interstitium of the heart reduces the differences in contrast signal between blood and myocardium such that the two compartments may null together or even be reversed and effusions may cause considerable ghosting artifact, although these both can be a strong clue to the underlying diagnosis (see Figure 4).^73,77,78^ Recently, the technique of equilibrium contrast CMR has demonstrated much higher extracellular myocardial volume in cardiac amyloid than any other measured disease.^79,80^ It is anticipated that accurate measurements of the expanded interstitium in amyloidosis will prove useful in serial quantification of cardiac amyloid burden.

### Radionuclide Imaging

**Figure 5.**
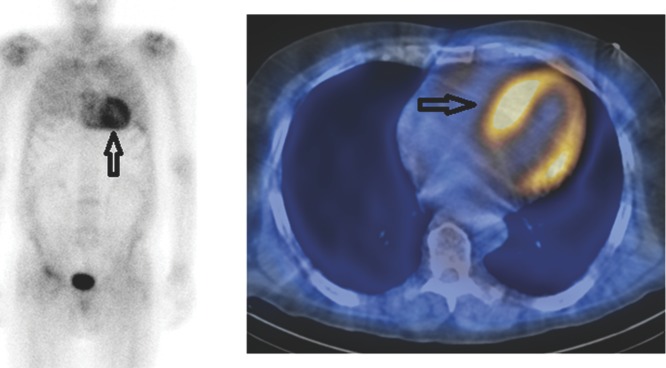
A positive 99mTc-DPD scan for TTR cardiac amyloid (left), showing uptake in the heart (arrow) and reduced bone uptake. The right-hand panel shows a fused CT/SPECT image showing myocardial uptake with greater uptake in the septum.

SAP component scintigraphy enables visceral amyloid deposits, including those in the liver, kidneys, spleen, adrenal glands, and bones, to be imaged serially in a specific and quantitative manner,^81^ but it does not adequately image the moving heart. Numerous case reports over the past 30 years have indicated that various commonly used diphosphonate bone-seeking radionuclide tracers occasionally localize to cardiac amyloid, and this approach has lately been investigated systematically. It transpires that ^99m^Tc-DPD, a particular tracer that has been little used of late for bone scintigraphy, appears to localize to cardiac amyloid deposits very sensitively, especially in patients with ATTR type (Figure 5). Indeed, asymptomatic cardiac ATTR deposits can be identified through ^99m^Tc-DPD scintigraphy at an early stage when echocardiography, serum cardiac biomarkers, and perhaps even CMR remain normal.^82^ By contrast, uptake of ^99m^Tc-DPD occurs in about one third of patients with cardiac AL amyloidosis, and ^99m^Tc-DPD-SPECT-CT can help to distinguish the two types.^82^ The sensitivity of DPD scintigraphy for detecting cardiac amyloidosis of TTR type would appear to have considerable potential for diagnosis and screening.^83^

### Endomyocardial Biopsy

**Figure 6.**
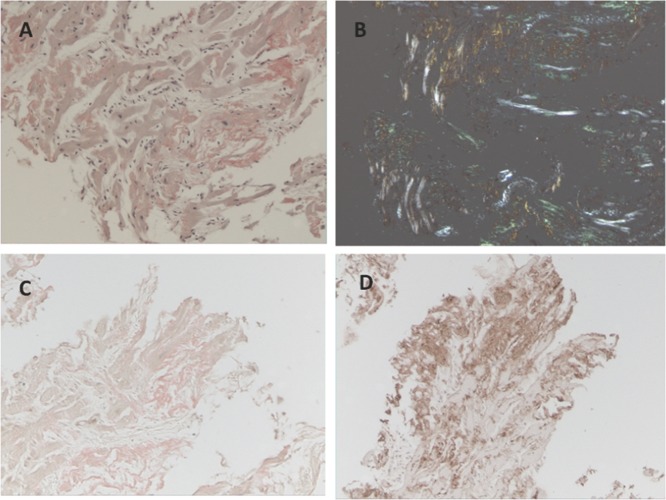
An endomyocardial biopsy of a patient with cardiac AL amyloidosis stained as follows: (A) Congo red only; (B) Apple-green birefringence under polarized light; (C) Congo red with lambda overlay (negative); (D) Congo red with kappa overlay (positive).

Endomyocardial biopsy has been considered to be the gold standard for demonstrating cardiac amyloid deposition.^84^ Although cardiac involvement can reasonably be inferred in a patient with proven systemic amyloidosis through a combination of clinical features, ECG, echocardiography, and biomarkers, etc, endomyocardial biopsy is required when suspected cardiac amyloidosis is an isolated feature or when the cardiac amyloid fibril type cannot be identified by other means. In practice, endomyocardial biopsies are most commonly required to differentiate between AL and ATTR in older patients, some 5% of whom have a monoclonal gammopathy of undetermined significance.^85^ Endomyocardial biopsies should be considered in patients with a thickened left ventricle by echocardiography where hypertension, valvular disease, and a family history of hypertrophic cardiomyopathy have been excluded, particularly if the patient is young. Complications such as perforation remain a small but real risk and may not be well tolerated in restrictive cardiomyopathy.^86,87^ The presence of amyloid deposition should be confirmed by Congo red staining, and immunohistochemistry can usefully identify fibril type in about 60% to 70% cases (see Figure 6). Electron microscopy to confirm or refute the presence of amyloid fibrils has an occasional role when Congo red stains fail to produce definitive results.^88,89^ Proteomic typing of amyloid by mass spectrometry using tiny samples obtained through laser capture microdissection of tissue sections usually provides definitive results^90^ and is critical when immunohistochemistry has not done so.

## Treatment

Cardiac amyloidosis in general has a poor prognosis, but this differs according to amyloid type and availability and response to therapy. Treatment may be classified as follows: supportive therapy (ie, modified heart-failure treatment including device therapy); therapies that suppress production of the respective amyloid fibril precursor protein (eg, chemotherapy in AL amyloidosis); and novel strategies to inhibit amyloid fibril formation or to directly target the amyloid deposits or stabilize the precursor protein (especially in ATTR with drugs such as tafamidis or diflunisal). Cardiac transplantation, although rarely feasible, can be very successful in carefully selected patients.

### Supportive Treatment

Standard heart-failure therapy may be of limited benefit or even detrimental in cardiac amyloidosis. There is scant evidence for the use (or not) of angiotensin-converting enzyme inhibitors, angiotensin receptor blockers, or β-blockers. These may be poorly tolerated and may worsen postural hypotension or renal function. Restrictive cardiomyopathy leads to a heart-rate–dependent cardiac output in some cases, and some such patients may find difficulty in tolerating β-blockers. Digitalis and calcium channel blockers may be selectively concentrated in amyloidotic tissue and are relatively contraindicated on grounds of increased toxicity,^91–93^ especially the latter, which can lead to rapid worsening. Careful monitoring is needed to avoid significant drug interactions, for example, β-blockers with thalidomide used in chemotherapy of AL amyloidosis causing bradycardia.^94^

Maintenance of adequate filling pressures is vital because of the restrictive physiology, balancing peripheral edema and renal impairment with salt/water restriction and judicious use of diuretics. Patient education and participation, ideally with backup from a heart failure team, are critical to successful management. Contrary to standard heart failure management, maintenance of adequate blood pressure with an α-agonist such as midodrine may permit higher doses of loop diuretics, especially in patients with autonomic neuropathy.^95^

### Device Therapy

Pacemakers or implantable cardioverter defibrillators may not prevent sudden cardiac death, because this is thought to often be due to electromechanical dissociation.^96^ In the absence of evidence, pacing indications remain within current standard guidelines. High defibrillator thresholds may be encountered, and the benefits of such devices remain uncertain.^96–98^ Biventricular pacing appears to play little role but may be the ideal pacing option to avoid decompensation of the stiffened ventricle as a result of induced dyssynchrony from right ventricular pacing.^99^

## Amyloid-Specific Treatment

### Reducing Amyloid Fibril Precursor Protein Production

Treatment of amyloidosis is currently based on the concept of reducing the supply of the respective amyloid fibril precursor protein. In AL amyloidosis, therapy is directed toward the clonal plasma cells using either cyclical combination chemotherapy or high-dose therapy with autologous stem cell transplantation. Most chemotherapy regimes in AL amyloidosis comprise dexamethasone combined with an alkylator (oral melphalan or others). Addition of thalidomide, for example, in the risk-adapted cyclophosphamide, thalidomide, and dexamethasone regime used widely in the United Kingdom, improves response rates but probably at cost of greater toxicity.^100^ Dexamethasone, although a very useful agent in all patients with AL amyloidosis, including those with cardiac involvement, has to be used with great caution in patients with cardiac amyloidosis because of a high risk of fluid overload in absence of adequate and rapid changes to diuretic therapy. Close coordination between the treating hematology and cardiology teams is critical to steer the patient successfully through the treatment course.^101^ High-dose melphalan followed by autologous stem-cell transplantation is generally contraindicated in patients with advanced cardiac amyloidosis. Although it has been argued that autologous stem cell transplantation is the best treatment for suitable patients,^102–104^ its role in the era of novel agents, discussed below, is less certain.

The newer treatment options include bortezomib (a proteosome inhibitor)^105^ and the newer immunomodulatory drugs lenalidomide and pomalidomide. Bortezomib combinations appear to be especially efficient in amyloidosis with high rates of near-complete clonal responses, which appear to translate into early cardiac responses.^106–108^ Phase II (bortezomib in combination with cyclophosphamide or doxorubicin) and phase III (bortezomib, melphalan, and dexamethasone compared to melphalan and dexamethasone as front-line treatment) trials are underway.

AA amyloidosis is the only other type of amyloidosis in which production of the fibril precursor protein can be effectively suppressed by currently available therapies. Anti-inflammatory therapies, such as anti-tumor necrosis factor agents in rheumatoid arthritis, can substantially suppress serum amyloid A protein production, but very little experience has been obtained regarding cardiac involvement, which is very rare in this particular type of amyloidosis.

TTR is produced almost exclusively in the liver, and TTR amyloidosis has lately become a focus for novel drug developments aimed at reducing production of TTR through silencing RNA and antisense oligonucleotide therapies. ALN-TTR01, a systemically delivered silencing RNA therapeutic,^109^ is already in phase I clinical trial. Liver transplantation has been used as a treatment for variant ATTR for 20 years, to remove genetically variant TTR from the plasma. Although this is a successful approach in ATTR Val30Met, it has had disappointing results in patients with other ATTR variants, which often involve the heart. The procedure commonly results in progressive cardiac amyloidosis through ongoing accumulation of wild-type TTR on the existing template of variant TTR amyloid.^110^ The role of liver transplantation in non-Val30Met–associated hereditary TTR amyloidosis thus remains very uncertain.

### Inhibition of Amyloid Formation

Amyloid fibril formation involves massive conformational transformation of the respective precursor protein into a completely different form with predominant β-sheet structure. The hypothesis that this conversion might be inhibited by stabilizing the fibril precursor protein through specific binding to a pharmaceutical has lately been explored in TTR amyloidosis. A key step in TTR amyloid fibril formation is the dissociation of the normal TTR tetramer into monomeric species that can autoaggregate in a misfolded form. In vitro studies identified that diflunisal, a now little used nonsteroidal anti-inflammatory analgesic, is bound by TTR in plasma, and that this enhances the stability of the normal soluble structure of the protein.^111,112^ Studies of diflunisal in ATTR are in progress. Tafamidis is a new compound without anti-inflammatory analgesic properties that has a similar mechanism of action. Tafamidis has just been licensed for neuropathic ATTR, but its role in cardiac amyloidosis remains uncertain, and clinical trial results are eagerly awaited. Higher-affinity “superstabilizers” are also in development.^113^

Eprodisate is a negatively charged, sulfonated molecule with similarities to heparan sulfate, which is being pursued as a treatment for AA amyloidosis. Eprodisate is thought to inhibit the pro-amyloidogenic interactions of glycosaminoglycans with SAA during fibril formation in AA amyloidosis. A phase III trial showed benefits in terms of progression of AA-amyloid–associated renal dysfunction,^114^ and further studies are currently being conducted.

### Targeting Amyloid Deposits by Immunotherapy

Amyloid deposits are remarkably stable, but the body evidently has some limited capacity to remove them. After treatment that prevents the production of new amyloid, for example, successful chemotherapy in AL type amyloid deposits are gradually mobilized in the majority of patients, though at different rates in different organs and different individuals. Unfortunately clearance of amyloid is especially slow in the heart, and echocardiographic evidence of improvement is rare, even over several years. The concept of passive immunotherapy to enhance clearance of amyloid has proved successful in experimental models and is variously now in clinical development.

The challenge of developing a therapeutic monoclonal antibody that is reactive with all types of amyloid has lately been addressed by targeting SAP because this is a universal constituent of all amyloid deposits and an excellent immunogen. Anti-SAP antibody treatment is clinically feasible because circulating human SAP can be depleted in patients by the bis-d-proline compound CPHPC, thereby enabling injected anti-SAP antibodies to reach residual SAP in the amyloid deposits.^3^ The unprecedented capacity of this novel combined therapy to eliminate amyloid deposits in mice is encouraging and should be applicable to all forms of human systemic and local amyloidosis.

### Cardiac Transplantation in Amyloidosis

Cardiac transplantation has played a disappointingly small role, because of the multisystem nature of amyloidosis, advanced age, treatment-related complications, and rapid disease progression. Further, patients with AL amyloidosis must be deemed likely to be sufficiently fit to undergo chemotherapy afterward, to address the underlying bone marrow disorder. As a result, only a few dozen cardiac transplantations have ever been performed for amyloidosis. However, the long-term outcome can be good in highly selected patients with AL amyloidosis.^115^ Cardiac transplantation followed by successful peripheral blood autologous stem cell transplantation was associated with better survival in selected patients, as reported^116^ from most major amyloidosis units in the United Kingdom,^115^ France,^117^ Germany,^118^ and the United States.^119^ A suitable patient with AL amyloidosis is likely to be young (<60 years); to have isolated Mayo stage III cardiac amyloidosis, NYHA III or IV symptoms after adequate diuretics, good renal/liver function, no significant autonomic neuropathy, and low-level bone marrow plasmacytosis; and to be eligible for autologous stem cell transplantation after the heart transplantation. Even in such patients, outcomes are probably inferior to other indications.^120^ For variant ATTR, combined cardiac and liver transplantation has been performed in a few dozen cases throughout the world.^115,116,121,122^ Although most patients with wild-type ATTR are too elderly for cardiac transplantation, the absence of extracardiac involvement renders younger patients with wild-type ATTR excellent candidates. The 2 patients with wild-type ATTR who presented to our center before age 60 survived 10 and 20 years, respectively, after cardiac transplantation.

## Conclusion

Cardiac amyloidosis remains challenging to diagnose and to treat. Key “red flags” that should raise suspicion include clinical features indicating multisystem disease and concentric LV thickening on echocardiography in the absence of increased voltage on ECG; the pattern of gadolinium enhancement on CMR appears to be very characteristic. Confirmation of amyloid type is now possible in most cases through a combination of immunohistochemistry, DNA analysis, and proteomics. Unlike other causes of heart failure, supportive treatment is mainly focused on diuretics therapy. Although developments in chemotherapy have greatly improved the outlook in AL amyloidosis, the prognosis of patients with advanced cardiac involvement remains very poor. Senile cardiac amyloidosis is probably greatly underdiagnosed, but CMR and DPD scintigraphy show great potential to address this unmet need in the aging population. A variety of novel specific therapies are on the near horizon, with potential to both inhibit new amyloid formation and enhance clearance of existing deposits.

## References

[1] Falk RH Diagnosis and management of the cardiac amyloidoses. Circulation. 2005;112:2047-2060 16186440 10.1161/CIRCULATIONAHA.104.489187

[2] Gutierrez PS Fernandes F Mady C Higuchi Mde L Clinical, electrocardiographic and echocardiographic findings in significant cardiac amyloidosis detected only at necropsy: comparison with cases diagnosed in life. Arq Bras Cardiol. 2008;90:191-196 18392399 10.1590/s0066-782x2008000300009

[3] Bodin K Ellmerich S Kahan MC Tennent GA Loesch A Gilbertson JA Hutchinson WL Mangione PP Gallimore JR Millar DJ Minogue S Dhillon AP Taylor GW Bradwell AR Petrie A Gillmore JD Bellotti V Botto M Hawkins PN Pepys MB Antibodies to human serum amyloid P component eliminate visceral amyloid deposits. Nature. 2010;468:93-97 20962779 10.1038/nature09494 PMC2975378

[4] Westermark P Benson MD Buxbaum JN Cohen AS Frangione B lkeda S Masters CL Merlini G Saraiva MJ Sipe JD A primer of amyloid nomenclature. Amyloid. 2007;14:179-183 17701465 10.1080/13506120701460923

[5] Sipe JD Benson MD Buxbaum JN Ikeda S Merlini G Saraiva MJ Westermark P Amyloid fibril protein nomenclature: 2010 recommendations from the nomenclature committee of the International Society of Amyloidosis. Amyloid. 2010;17:101-104 21039326 10.3109/13506129.2010.526812

[6] Kisilevsky R The relation of proteoglycans, serum amyloid P and apo E to amyloidosis current status, 2000. Amyloid. 2000;7:23-25 10842701 10.3109/13506120009146820

[7] Woodrow SI Stewart RJ Kisilevsky R Gore J Young ID Experimental AA amyloidogenesis is associated with differential expression of extracellular matrix genes. Amyloid. 1999;6:22-30 10211408 10.3109/13506129908993284

[8] Westermark GT Norling B Westermark P Fibronectin and basement membrane components in renal amyloid deposits in patients with primary and secondary amyloidosis. Clin Exp Immunol. 1991;86:150-156 1914228 10.1111/j.1365-2249.1991.tb05788.x PMC1554156

[9] Falk RH Dubrey SW Amyloid heart disease. Prog Cardiovasc Dis. 2010;52:347-361 20109604 10.1016/j.pcad.2009.11.007

[10] Brodarick S Paine R Higa E Carmichael KA Pericardial tamponade, a new complication of amyloid heart disease. Am J Med. 1982;73:133-135 7091168 10.1016/0002-9343(82)90939-1

[11] Navarro JF Rivera M Ortuno J Cardiac tamponade as presentation of systemic amyloidosis. Int J Cardiol. 1992;36:107-108 1428240 10.1016/0167-5273(92)90115-j

[12] Chamarthi B Dubrey SW Cha K Skinner M Falk RH Features and prognosis of exertional syncope in light-chain associated AL cardiac amyloidosis. Am J Cardiol. 1997;80:1242-1245 9359565 10.1016/s0002-9149(97)00653-x

[13] Padfield GJ Maclay JD Macroglossia and complete heart block in a woman with multiple myeloma. QJM. 2010;103:271-272 19713192 10.1093/qjmed/hcp124

[14] Payne CE Usher BW Atrioventicular block in familial amyloidosis: revisiting an old debate. J SC Med Assoc. 2007;103:119-122 18333575

[15] Ridolfi RL Bulkley BH Hutchins GM The conduction system in cardiac amyloidosis: clinical and pathologic features of 23 patients. Am J Med. 1977;62:677-686 871125 10.1016/0002-9343(77)90870-1

[16] Velazquez-Cecena JL Lubell DL Nagajothi N Al-Masri H Siddiqui M Khosla S Syncope from dynamic left ventricular outflow tract obstruction simulating hypertrophic cardiomyopathy in a patient with primary AL-type amyloid heart disease. Tex Heart Inst J. 2009;36:50-54 19436787 PMC2676515

[17] Dinwoodey DL Skinner M Maron MS Davidoff R Ruberg FL Light-chain amyloidosis with echocardiographic features of hypertrophic cardiomyopathy. Am J Cardiol. 2008;101:674-676 18308019 10.1016/j.amjcard.2007.10.031

[18] Dubrey SW Cha K Anderson J Chamarthi B Reisinger J Skinner M Falk RH The clinical features of immunoglobulin light-chain (AL) amyloidosis with heart involvement. QJM. 1998;91:141-157 9578896 10.1093/qjmed/91.2.141

[19] Morner S Hellman U Suhr OB Kazzam E Waldenstrom A Amyloid heart disease mimicking hypertrophic cardiomyopathy. J Intern Med. 2005;258:225-230 16115295 10.1111/j.1365-2796.2005.01522.x

[20] Mueller PS Edwards WD Gertz MA Symptomatic ischemic heart disease resulting from obstructive intramural coronary amyloidosis. Am J Med. 2000;109:181-188 10974179 10.1016/s0002-9343(00)00471-x

[21] AlSuwaidi J Velianou JL Gertz MA Cannon RO Higano ST Holmes DR Lerman A Systemic amyloidosis presenting with angina pectoris. Ann Intern Med. 1999;131:838-841 10610629 10.7326/0003-4819-131-11-199912070-00007

[22] Feng D Edwards WD Oh JK Chandrasekaran K Grogan M Martinez MW Syed IS Hughes DA Lust JA Jaffe AS Gertz MA Klarich KW Intracardiac thrombosis and embolism in patients with cardiac amyloidosis. Circulation. 2007;116:2420-2426 17984380 10.1161/CIRCULATIONAHA.107.697763

[23] Zubkov AY Rabinstein AA Dispenzieri A Wijdicks EF Primary systemic amyloidosis with ischemic stroke as a presenting complication. Neurology. 2007;69:1136-1141 17846413 10.1212/01.wnl.0000276951.39112.2b

[24] Poels MM Ikram MA van der Lugt A Hofman A Krestin GP Breteler MM Vernooij MW Incidence of cerebral microbleeds in the general population: the Rotterdam Scan Study. Stroke. 2011;42:656-661 21307170 10.1161/STROKEAHA.110.607184

[25] Kyle RA Gertz MA Primary systemic amyloidosis: clinical and laboratory features in 474 cases. Semin Hematol. 1995;32:45-59 7878478

[26] Merlini G Stone MJ Dangerous small B-cell clones. Blood. 2006;108:2520-2530 16794250 10.1182/blood-2006-03-001164

[27] Dispenzieri A Gertz MA Kyle RA Lacy MQ Burritt MF Therneau TM Greipp PR Witzig TE Lust JA Rajkumar SV Fonseca R Zeldenrust SR McGregor CG Jaffe AS Serum cardiac troponins and N-terminal pro–brain natriuretic peptide: a staging system for primary systemic amyloidosis. J Clin Oncol. 2004;22:3751-3757 15365071 10.1200/JCO.2004.03.029

[28] Jacobson DR Pastore RD Yaghoubian R Kane I Gallo G Buck FS Buxbaum JM Variant-sequence transthyretin (isoleucine 122) in late-onset cardiac amyloidosis in black Americans. N Engl J Med. 1997;336:466-473 9017939 10.1056/NEJM199702133360703

[29] Falk RH The neglected entity of familial cardiac amyloidosis in African Americans. Ethn Dis. 2002;12:141-143 11913602

[30] Merlini G Bellotti V Molecular mechanisms of amyloidosis. N Engl J Med. 2003;349:583-596 12904524 10.1056/NEJMra023144

[31] Dubrey SW Hawkins PN Falk RH Amyloid diseases of the heart: assessment, diagnosis, and referral. Heart. 2011;97:75-84 21148582 10.1136/hrt.2009.190405

[32] Westermark P Sletten K Johansson B Cornwell GG,3rd Fibril in senile systemic amyloidosis is derived from normal transthyretin. Proc Natl Acad Sci USA. 1990;87:2843-2845 2320592 10.1073/pnas.87.7.2843 PMC53787

[33] Ng B Connors LH Davidoff R Skinner M Falk RH Senile systemic amyloidosis presenting with heart failure: a comparison with light chain-associated amyloidosis. Arch Intern Med. 2005;165:1425-1429 15983293 10.1001/archinte.165.12.1425

[34] Connors LH Lim A Prokaeva T Roskens VA Costello CE Tabulation of human transthyretin (TTR) variants, 2003. Amyloid. 2003;10:160-184 14640030 10.3109/13506120308998998

[35] Dubrey SW Falk RH Amyloid heart disease. Br J Hosp Med (Lond). 2010;71:76-82 20220694 10.12968/hmed.2010.71.2.46484

[36] Tanskanen M Peuralinna T Polvikoski T Notkola IL Sulkava R Hardy J Singleton A Kiuru-Enari S Paetau A Tienari PJ Myllykangas L Senile systemic amyloidosis affects 25% of the very aged and associates with genetic variation in alpha2-macroglobulin and tau: a population-based autopsy study. Ann Med. 2008;40:232-239 18382889 10.1080/07853890701842988

[37] Ogiwara F Koyama J Ikeda S Kinoshita O Falk RH Comparison of the strain Doppler echocardiographic features of familial amyloid polyneuropathy (FAP) and light-chain amyloidosis. Am J Cardiol. 2005;95:538-540 15695150 10.1016/j.amjcard.2004.10.029

[38] Ariyarajah V Steiner I Hajkova P Khadem A Kvasnicka J Apiyasawat S Spodick DH The association of atrial tachyarrhythmias with isolated atrial amyloid disease: preliminary observations in autopsied heart specimens. Cardiology. 2009;113:132-137 19039221 10.1159/000177950

[39] Kaye GC Butler MG d'Ardenne AJ Edmondson SJ Camm AJ Slavin G Isolated atrial amyloid contains atrial natriuretic peptide: a report of six cases. Br Heart J. 1986;56:317-320 2945573 10.1136/hrt.56.4.317 PMC1236864

[40] Steiner I The prevalence of isolated atrial amyloid. J Pathol. 1987;153:395-398 3430237 10.1002/path.1711530413

[41] Kristen AV Schnabel PA Winter B Helmke BM Longerich T Hardt S Koch A Sack FU Katus HA Linke RP Dengler TJ High prevalence of amyloid in 150 surgically removed heart valves—a comparison of histological and clinical data reveals a correlation to atheroinflammatory conditions. Cardiovasc Pathol. 2010;19:228-235 19502085 10.1016/j.carpath.2009.04.005

[42] Iqbal S Reehana S Lawrence D Unique type of isolated cardiac valvular amyloidosis. J Cardiothorac Surg. 2006;1:38 17062163 10.1186/1749-8090-1-38 PMC1634846

[43] Cheng ZW Tian Z Kang L Chen TB Fang LG Cheng KA Zeng Y Fang Q Electrocardiographic and echocardiographic features of patients with primary cardiac amyloidosis. Zhonghua Xin Xue Guan Bing Za Zhi. 2010;38:606-609 21055282

[44] Murtagh B Hammill SC Gertz MA Kyle RA Tajik AJ Grogan M Electrocardiographic findings in primary systemic amyloidosis and biopsy-proven cardiac involvement. Am J Cardiol. 2005;95:535-537 15695149 10.1016/j.amjcard.2004.10.028

[45] Rapezzi C Merlini G Quarta CC Riva L Longhi S Leone O Salvi F Ciliberti P Pastorelli F Biagini E Coccolo F Cooke RM Bacchi-Reggiani L Sangiorgi D Ferlini A Cavo M Zamagni E Fonte ML Palladini G Salinaro F Musca F Obici L Branzi A Perlini S Systemic cardiac amyloidoses: disease profiles and clinical courses of the 3 main types. Circulation. 2009;120:1203-1212 19752327 10.1161/CIRCULATIONAHA.108.843334

[46] Takigawa M Hashimura K Ishibashi-Ueda H Yamada N Kiso K Nanasato M Yoshida Y Hirayama H Annual electrocardiograms consistent with silent progression of cardiac involvement in sporadic familial amyloid polyneuropathy: a case report. Intern Med. 2010;49:139-144 20075578 10.2169/internalmedicine.49.2703

[47] Pinney JGJ Lachmann H Wechalekar A Gibbs S Banypersad SM Dungu J Rannigan L Collins E McCarthy C Hawkins P Whelan C Disturbances of Cardiac Rhythm in AL Amyloidosis. 13th International Myeloma Workshop (Abstract). 2011 426

[48] Kristen AV Perz JB Schonland SO Hansen A Hegenbart U Sack FU Goldschmidt H Katus HA Dengler TJ Rapid progression of left ventricular wall thickness predicts mortality in cardiac light-chain amyloidosis. J Heart Lung Transplant. 2007;26:1313-1319 18096484 10.1016/j.healun.2007.09.014

[49] Koyama J Falk RH Prognostic significance of strain Doppler imaging in light-chain amyloidosis. JACC Cardiovasc Imaging. 2010;3:333-342 20394893 10.1016/j.jcmg.2009.11.013

[50] Migrino RQ Mareedu RK Eastwood D Bowers M Harmann L Hari P Left ventricular ejection time on echocardiography predicts long-term mortality in light chain amyloidosis. J Am Soc Echocardiogr. 2009;22:1396-1402 19880277 10.1016/j.echo.2009.09.012 PMC2787973

[51] Patel AR Dubrey SW Mendes LA Skinner M Cupples A Falk RH Davidoff R Right ventricular dilation in primary amyloidosis: an independent predictor of survival. Am J Cardiol. 1997;80:486-492 9285663 10.1016/s0002-9149(97)00400-1

[52] Porciani MC Lilli A Perfetto F Capelli F Massimiliano Rao C Del Pace S Ciaccheri M Castelli G Tarquini R Romagnani L Pastorini T Padeletti L Bergesio F Tissue Doppler and strain imaging: a new tool for early detection of cardiac amyloidosis. Amyloid. 2009;16:63-70 20536397 10.1080/13506120902879681

[53] Tsang W Lang RM Echocardiographic evaluation of cardiac amyloid. Curr Cardiol Rep. 2010;12:272-276 20424972 10.1007/s11886-010-0108-7

[54] Bellavia D Pellikka PA Abraham TP AL-Zahrani GB Dispenzieri A Oh JK Bailey KR Wood CM Lacy MQ Miyazaki C Miller FA Evidence of impaired left ventricular systolic function by Doppler myocardial imaging in patients with systemic amyloidosis and no evidence of cardiac involvement by standard two-dimensional and Doppler echocardiography. Am J Cardiol. 2008;101:1039-1045 18359328 10.1016/j.amjcard.2007.11.047

[55] Park SJ Miyazaki C Bruce CJ Ommen S Miller FA Oh JK Left ventricular torsion by two-dimensional speckle tracking echocardiography in patients with diastolic dysfunction and normal ejection fraction. J Am Soc Echocardiogr. 2008;21:1129-1137 18486443 10.1016/j.echo.2008.04.002

[56] Porciani MC Cappelli F Perfetto F Ciaccheri M Castelli G Ricceri I Chiostri M Franco B Padeletti L Rotational mechanics of the left ventricle in AL amyloidosis. Echocardiography. 2010;27:1061-1068 21039810 10.1111/j.1540-8175.2010.01199.x

[57] Kusunose K Yamada H Iwase T Nishio S Tomita N Niki T Yamaguchi K Koshiba K Taketani Y Soeki T Wakatsuki T Akaike M Shoichiro T Harada M Kagawa N Kudo E Sata M Images in cardiovascular medicine: cardiac magnetic resonance imaging and 2-dimensional speckle tracking echocardiography in secondary cardiac amyloidosis. Circ J. 2010;74:1494-496 20501956 10.1253/circj.cj-10-0141

[58] Sun JP Stewart WJ Yang XS Donnell RO Leon AR Felner JM Thomas JD Merlino JD Differentiation of hypertrophic cardiomyopathy and cardiac amyloidosis from other causes of ventricular wall thickening by two-dimensional strain imaging echocardiography. Am J Cardiol. 2009;103:411-415 19166699 10.1016/j.amjcard.2008.09.102

[59] Abdelmoneim SS Bernier M Bellavia D Syed IS Mankad SV Chandrasekaran K Pellikka PA Mulvagh SL Myocardial contrast echocardiography in biopsy-proven primary cardiac amyloidosis. Eur J Echocardiogr. 2008;9:338-341 18490333 10.1093/ejechocard/jen017

[60] Feng D Syed IS Martinez M Oh JK Jaffe AS Grogan M Edwards WD Gertz MA Klarich KW Intracardiac thrombosis and anticoagulation therapy in cardiac amyloidosis. Circulation. 2009;119:2490-2497 19414641 10.1161/CIRCULATIONAHA.108.785014

[61] Palladini G Campana C Klersy C Balduini A Vadacca G Perfetti V Perlini S Obici L Ascari E d'Eril GM Moratti R Merlini G Serum N-terminal pro-brain natriuretic peptide is a sensitive marker of myocardial dysfunction in AL amyloidosis. Circulation. 2003;107:2440-2445 12719281 10.1161/01.CIR.0000068314.02595.B2

[62] Wechalekar AD Gillmore JD Wassef N Lachmann HJ Whelan C Hawkins PN Abnormal N-terminal fragment of brain natriuretic peptide in patients with light chain amyloidosis without cardiac involvement at presentation is a risk factor for development of cardiac amyloidosis. Haematologica. 2011;96:1079-80 21606171 10.3324/haematol.2011.040493 PMC3128232

[63] Takemura G Takatsu Y Doyama K Itoh H Saito Y Koshiji M Ando F Fujiwara T Nakao K Fujiwara H Expression of atrial and brain natriuretic peptides and their genes in hearts of patients with cardiac amyloidosis. J Am Coll Cardiol. 1998;31:754-765 9525543 10.1016/s0735-1097(98)00045-x

[64] Dispenzieri A Kyle RA Gertz MA Therneau TM Miller WL Chandrasekaran K McConnell JP Burritt MF Jaffe AS Survival in patients with primary systemic amyloidosis and raised serum cardiac troponins. Lancet. 2003;361:1787-1789 12781539 10.1016/S0140-6736(03)13396-X

[65] Palladini G Barassi A Klersy C Pacciolla R Milani P Sarais G Perlini S Albertini R Russo P Foli A Bragotti LZ Obici L Moratti L d'eril GV Merlini G The combination of high-sensitivity cardiac troponin T (hs-cTnT) at presentation and changes in N-terminal natriuretic peptide type B (NT-proBNP) after chemotherapy best predicts survival in AL amyloidosis. Blood. 2010;116:3426-3430 20644111 10.1182/blood-2010-05-286567

[66] Dispenzieri A Gertz MA Kyle RA Lacy MQ Burritt MF Therneau TM McConnell JP Litzow MR Gastineau DA Tefferi A Inwards DJ Micallef IN Ansell SM Poratta LF Elliott MA Hogan WJ Rajkumar SV Fonseca R Greipp PR Witzig TR Lust JA Zeldenrust SR Snow DS Hayman SR McGregor CG Jaffe AS Prognostication of survival using cardiac troponins and N-terminal pro-brain natriuretic peptide in patients with primary systemic amyloidosis undergoing peripheral blood stem cell transplantation. Blood. 2004;104:1881-1887 15044258 10.1182/blood-2004-01-0390

[67] Palladini G Russo P Nuvolone M Lavatelli F Perfetti V Obici L Merlini G Treatment with oral melphalan plus dexamethasone produces long-term remissions in AL amyloidosis. Blood. 2007;110:787-788 17606766 10.1182/blood-2007-02-076034

[68] Tapan U Seldin DC Finn KT Fennessey S Shelton A Zeldis JB Sanchorawala V Increases in B-type natriuretic peptide (BNP) during treatment with lenalidomide in AL amyloidosis. Blood. 2010;116:5071-5072 21127185 10.1182/blood-2010-09-305136

[69] Dispenzieri A Dingli D Kumar SK Rajkumar SV Lacy MQ Hayman S Buadi F Zeldenrust S Leung N Detweiler-Short K Lust JA Russell SJ Kyle RA Gertz MA Discordance between serum cardiac biomarker and immunoglobulin-free light-chain response in patients with immunoglobulin light-chain amyloidosis treated with immune modulatory drugs. Am J Hematol. 2010;85:757-759 20872958 10.1002/ajh.21822 PMC3691013

[70] Hosch W Bock M Libicher M Ley S Hegenbart U Dengler TJ Katus HA Kauczor HU Kauffmann GW Kristen AV MR-relaxometry of myocardial tissue: significant elevation of T1 and T2 relaxation times in cardiac amyloidosis. Invest Radiol. 2007;42:636-642 17700279 10.1097/RLI.0b013e318059e021

[71] Sparrow P Amirabadi A Sussman MS Paul N Merchant N Quantitative assessment of myocardial T2 relaxation times in cardiac amyloidosis. J Magn Reson Imaging. 2009;30:942-946 19780184 10.1002/jmri.21918

[72] Vogelsberg H Mahrholdt H Deluigi CC Yilmaz A Kispert EM Greulich S Kilngel K Kandolf R Sechtem U Cardiovascular magnetic resonance in clinically suspected cardiac amyloidosis: noninvasive imaging compared to endomyocardial biopsy. J Am Coll Cardiol. 2008;51:1022-1030 18325442 10.1016/j.jacc.2007.10.049

[73] Maceira AM Joshi J Prasad SK Moon JC Perugini E Harding I Sheppard MN Poole-Wilson PA Hawkins PN Pennell DJ Cardiovascular magnetic resonance in cardiac amyloidosis. Circulation. 2005;111:186-193 15630027 10.1161/01.CIR.0000152819.97857.9D

[74] Austin BA Tang WH Rodriguez ER Tan C Flamm SD Taylor DO Starling RC Desai MY Delayed hyper-enhancement magnetic resonance imaging provides incremental diagnostic and prognostic utility in suspected cardiac amyloidosis. JACC Cardiovasc Imaging. 2009;2:1369-1277 20083070 10.1016/j.jcmg.2009.08.008

[75] Syed IS Glockner JF Feng D Araoz PA Martinez MW Edwards WD Gertz MA Dispenzieri A Oh JK Bellavia D Tajik AJ Grogan M Role of cardiac magnetic resonance imaging in the detection of cardiac amyloidosis. JACC Cardiovasc Imaging. 2010;3:155-164 20159642 10.1016/j.jcmg.2009.09.023

[76] Di Bella G Minutoli F Mazzeo A Vita G Oreto G Carerj S Anfuso C Russo M Gaeta M MRI of cardiac involvement in transthyretin familial amyloid polyneuropathy. AJR Am J Roentgenol. 2010;195:W394-W399 21098170 10.2214/AJR.09.3721

[77] Maceira AM Prasad SK Hawkins PN Roughton M Pennell DJ Cardiovascular magnetic resonance and prognosis in cardiac amyloidosis. J Cardiovasc Magn Reson. 2008;10:54 19032744 10.1186/1532-429X-10-54 PMC2605441

[78] Sparrow PJ Merchant N Provost YL Doyle DJ Nguyen ET Paul NS CT and MR imaging findings in patients with acquired heart disease at risk for sudden cardiac death. Radiographics. 2009;29:805-823 19448117 10.1148/rg.293085715

[79] Flett AS Hayward MP Ashworth MT Hansen MS Taylor AM Elliott PM McGregor C Moon JC Equilibrium contrast cardiovascular magnetic resonance for the measurement of diffuse myocardial fibrosis: preliminary validation in humans. Circulation. 2010;122:138-144 20585010 10.1161/CIRCULATIONAHA.109.930636

[80] Banypersad SMSD Flett AS Gibbs S Pinney J Maestrini V White S Dungu J Hawkins PN Moon JC Cardiac involvement in cardiac AL amyloidosis as measured by equilibrium contrast cardiovascular magnetic resonance [abstract]. J Cardiovasc Magn Reson. 2012;14 Suppl 174

[81] Hawkins PN Lavender JP Pepys MB Evaluation of systemic amyloidosis by scintigraphy with 123I-labeled serum amyloid P component. N Engl J Med. 1990;323:508-513 2377176 10.1056/NEJM199008233230803

[82] Minutoli FGM Di Bella G Crisafulli C Murè G Militano V Brancati M Di Leo R Mazzeo A Baldari S Cardiac involvement in transthyretin familial amyloid polyneuropathy—comparison between 99mTc–DPD SPECT and magnetic resonance imaging. Eur Assoc Nuclear Med. 2010 252

[83] Perugini E Guidalotti PL Salvi F Cooke RM Pettinato C Riva L Leone O Farsad M Ciliberti P Bacchi-Reggiani L Fallani F Branzi A Rapezzi C Noninvasive etiologic diagnosis of cardiac amyloidosis using 99mTc-3,3-diphosphono-1,2-propanodicarboxylic acid scintigraphy. J Am Coll Cardiol. 2005;46:1076-1084 16168294 10.1016/j.jacc.2005.05.073

[84] Kieninger B Eriksson M Kandolf R Schnabel PA Schonland S Kristen AV Hegenbart U Lohse P Rocken C Amyloid in endomyocardial biopsies. Virchows Arch. 2010;456:523-532 20376481 10.1007/s00428-010-0909-5

[85] Benson MD Breall J Cummings OW Liepnieks JJ Biochemical characterisation of amyloid by endomyocardial biopsy. Amyloid. 2009;16:9-14 19291509 10.1080/13506120802676914

[86] Tian Z Zeng Y Cheng KA Gao P Zhao DC Cui QC Jiang XC Chen LF Fang Q Importance of endomyocardial biopsy in unexplained cardiomyopathy in China: a report of 53 consecutive patients. Chin Med J (Engl). 2010;123:864-870 20497679

[87] Sloan KP Bruce CJ Oh JK Rihal CS Complications of echocardiography-guided endomyocardial biopsy. J Am Soc Echocardiogr. 2009;22:324 e1–e4 19258180 10.1016/j.echo.2008.12.023

[88] Ardehali H Qasim A Cappola T Howard D Hruban R Hare JM Baughman KL Kasper EK Endomyocardial biopsy plays a role in diagnosing patients with unexplained cardiomyopathy. Am Heart J. 2004;147:919-923 15131552 10.1016/j.ahj.2003.09.020

[89] Gertz MA Grogan M Kyle RA Tajik AJ Endomyocardial biopsy-proven light chain amyloidosis (AL) without echocardiographic features of infiltrative cardiomyopathy. Am J Cardiol. 1997;80:93-95 9205031

[90] Vrana JA Gamez JD Madden BJ Theis JD Bergen HR 3rd Dogan A Classification of amyloidosis by laser microdissection and mass spectrometry-based proteomic analysis in clinical biopsy specimens. Blood. 2009;114:4957-4959 19797517 10.1182/blood-2009-07-230722

[91] Rubinow A Skinner M Cohen AS Digoxin sensitivity in amyloid cardiomyopathy. Circulation. 1981;63:1285-1288 7014028 10.1161/01.cir.63.6.1285

[92] Gertz MA Skinner M Connors LH Falk RH Cohen AS Kyle RA Selective binding of nifedipine to amyloid fibrils. Am J Cardiol. 1985;55:1646 4003315 10.1016/0002-9149(85)90996-8

[93] Pollak A Falk RH Left ventricular systolic dysfunction precipitated by verapamil in cardiac amyloidosis. Chest. 1993;104:618-620 8339658 10.1378/chest.104.2.618

[94] Yamaguchi T Syncope and sinus bradycardia from combined use of thalidomide and beta-blocker. Pharmacoepidemiol Drug Saf. 2008;17:1033-1035 18613249 10.1002/pds.1624

[95] Low PA Autonomic neuropathies. Curr Opin Neurol. 2002;15:605-609 12352004 10.1097/00019052-200210000-00011

[96] Kristen AV Dengler TJ Hegenbart U Schonland SO Goldschmidt H Sack FU Voss F Becker R Katus HA Bauer A Prophylactic implantation of cardioverter-defibrillator in patients with severe cardiac amyloidosis and high risk for sudden cardiac death. Heart Rhythm. 2008;5:235-240 18242546 10.1016/j.hrthm.2007.10.016

[97] Seethala S Jain S Ohori NP Monaco S Lacomis J Crock F Nemec J Focal monomorphic ventricular tachycardia as the first manifestation of amyloid cardiomyopathy. Indian Pacing Electrophysiol J. 2010;10:143-147 20234811 PMC2833238

[98] Dhoble A Khasnis A Olomu A Thakur R Cardiac amyloidosis treated with an implantable cardioverter defibrillator and subcutaneous array lead system: report of a case and literature review. Clin Cardiol. 2009;32:E63-E65 19455567 10.1002/clc.20389 PMC6653454

[99] Bellavia D Pellikka PA Abraham TP Al-Zahrani GB Dispenzieri A Oh JK Espinosa RE Scott CG Miyazaki C Miller FA ‘Hypersynchronisation’ by tissue velocity imaging in patients with cardiac amyloidosis. Heart. 2009;95:234-240 18474536 10.1136/hrt.2007.140343

[100] Wechalekar AD Goodman HJ Lachmann HJ Offer M Hawkins PN Gillmore JD Safety and efficacy of risk-adapted cyclophosphamide, thalidomide, and dexamethasone in systemic AL amyloidosis. Blood. 2007;109:457-464 16990593 10.1182/blood-2006-07-035352

[101] Dhodapkar MV Hussein MA Rasmussen E Solomon A Larson RA Crowley JJ Barlogie B Clinical efficacy of high-dose dexamethasone with maintenance dexamethasone/alpha interferon in patients with primary systemic amyloidosis: results of United States Intergroup Trial Southwest Oncology Group (SWOG) S9628. Blood. 2004;104:3520-3526 15308571 10.1182/blood-2004-05-1924

[102] Cohen AD Zhou P Chou J Teruya-Feldstein J Reich L Hassoun H Levine B Filippa DA Riedel E Kewalramani T Stubblefield MD Fleisher M Nimer S Comenzo RL Risk-adapted autologous stem cell transplantation with adjuvant dexamethasone +/- thalidomide for systemic light-chain amyloidosis: results of a phase II trial. Br J Haematol. 2007;139:224-233 17897298 10.1111/j.1365-2141.2007.06783.x

[103] Comenzo RL Managing systemic light-chain amyloidosis. J Natl Compr Canc Netw. 2007;5:179-187 17335687 10.6004/jnccn.2007.0018

[104] Wechalekar AD Hawkins PN Gillmore JD Perspectives in treatment of AL amyloidosis. Br J Haematol. 2008;140:365-377 18162121 10.1111/j.1365-2141.2007.06936.x

[105] Oliva L Palladini G Cerruti F Assessing proteostasis and proteasome stress in light chain amyloidosis. Blood (abstracts). 2010;116:3992

[106] Mikhael JR Schuster SR Jimenez-Zepeda VH The combination of cyclophosphamide-bortezomib-dexamethasone (CYBOR-D) is a highly effective and well tolerated regimen that produces rapid and complete hematological response in patients with AL amyloidosis. ASH Annu Meet Abstr. 2010;116:3063

[107] Wechalekar AD Kastritis E Merlini G A European collaborative study of treatment outcomes in 428 patients with systemic AL amyloidosis. ASH Annu Meet Abstr. 2010;116:988

[108] Zonder JA Sanchorawala V Snyder RM Melphalan and dexamethasone plus bortezomib induces hematologic and organ responses in AL-amyloidosis with tolerable neurotoxicity. ASH Annu Meet Abstr. 2009;114:746

[109] Benson MD Kluve-Beckerman B Zeldenrust SR Siesky AM Bodenmiller DM Showalter AD Sloop KW Targeted suppression of an amyloidogenic transthyretin with antisense oligonucleotides. Muscle Nerve. 2006;33:609-618 16421881 10.1002/mus.20503

[110] Ihse E Suhr OB Hellman U Westermark P Variation in amount of wild-type transthyretin in different fibril and tissue types in ATTR amyloidosis. J Mol Med. 2011;89:171-180 21107516 10.1007/s00109-010-0695-1 PMC3022153

[111] Sekijima Y Dendle MA Kelly JW Orally administered diflunisal stabilizes transthyretin against dissociation required for amyloidogenesis. Amyloid. 2006;13:236-249 17107884 10.1080/13506120600960882

[112] Tojo K Sekijima Y Kelly JW Ikeda S Diflunisal stabilizes familial amyloid polyneuropathy-associated transthyretin variant tetramers in serum against dissociation required for amyloidogenesis. Neurosci Res. 2006;56:441-449 17028027 10.1016/j.neures.2006.08.014

[113] Kolstoe SE Mangione PP Bellotti V Taylor GW Tennent GA Deroo S Morrison AJ Cobb AJ Coyne A McCammon MG Warner TD Mitchell J Gill R Smith MD Ley SV Robinson CV Wood SP Pepys MB Trapping of palindromic ligands within native transthyretin prevents amyloid formation. Proc Natl Acad Sci USA. 2010;107:20483-20488 21059958 10.1073/pnas.1008255107 PMC2996680

[114] Dember LM Hawkins PN Hazenberg BP Gorevic PD Merlini G Butrimiene I Livneh A Lesnyak O Puechal X Lachmann HJ Obici L Balshaw R Garceau D Hauck W Skinner M Eprodisate for the treatment of renal disease in AA amyloidosis. N Engl J Med. 2007;356:2349-2360 17554116 10.1056/NEJMoa065644

[115] Sattianayagam PT Gibbs SD Pinney JH Wechalekar AD Lachmann HJ Whelan CJ Gilbertson JA Hawkins PN Gillmore JD Solid organ transplantation in AL amyloidosis. Am J Transplant. 2010;10:2124-31 20883547 10.1111/j.1600-6143.2010.03227.x

[116] Gillmore JD Goodman HJ Lachmann HJ Offer M Wechalekar AD Joshi J Pepys MB Hawkins PN Sequential heart and autologous stem cell transplantation for systemic AL amyloidosis. Blood. 2006;107:1227-1229 16210334 10.1182/blood-2005-08-3253

[117] Mignot A Varnous S Redonnet M Jaccard A Epailly E Vermes E Boissonnat P Gandjbakhch I Herpin D Touchard G Bridoux F Heart transplantation in systemic (AL) amyloidosis: a retrospective study of eight French patients. Arch Cardiovasc Dis. 2008;101:523-532 19041836 10.1016/j.acvd.2008.06.018

[118] Sack FU Kristen A Goldschmidt H Schnabel PA Dengler T Koch A Karck M Treatment options for severe cardiac amyloidosis: heart transplantation combined with chemotherapy and stem cell transplantation for patients with AL-amyloidosis and heart and liver transplantation for patients with ATTR-amyloidosis. Eur J Cardiothorac Surg. 2008;33:257-262 18096396 10.1016/j.ejcts.2007.10.025

[119] Dey BR Chung SS Spitzer TR Zheng H Macgillivray TE Seldin DC McAfee S Ballen K Attar E Want T Shin J Newton-Cheh C Moore S Sanchorawala V Skinner M Madsen JC Semigran MJ Cardiac transplantation followed by dose-intensive melphalan and autologous stem-cell transplantation for light chain amyloidosis and heart failure. Transplantation. 2010;90:905-911 20733534 10.1097/TP.0b013e3181f10edb PMC2964067

[120] Dubrey SW Burke MM Hawkins PN Banner NR Cardiac transplantation for amyloid heart disease: the United Kingdom experience. J Heart Lung Transplant. 2004;23:1142-1153 15477107 10.1016/j.healun.2003.08.027

[121] Barreiros AP Post F Hoppe-Lotichius M Linke RP Vahl CF Schafers HJ Galle PR Otto G Liver transplantation and combined liver-heart transplantation in patients with familial amyloid polyneuropathy: a single-center experience. Liver Transpl. 2010;16:314-323 20209591 10.1002/lt.21996

[122] Hamour IM Lachmann HJ Goodman HJ Petrou M Burke MM Hawkins PN Banner NR Heart transplantation for homozygous familial transthyretin (TTR) V122I cardiac amyloidosis. Am J Transplant. 2008;8:1056-1059 18318779 10.1111/j.1600-6143.2008.02162.x

